# A New Personalized Cooling Protocol to Activate Brown Adipose Tissue in Young Adults

**DOI:** 10.3389/fphys.2017.00863

**Published:** 2017-11-02

**Authors:** Borja Martinez-Tellez, Guillermo Sanchez-Delgado, Yolanda Garcia-Rivero, Juan M. A. Alcantara, Wendy D. Martinez-Avila, Maria V. Muñoz-Hernandez, Josune Olza, Mariëtte R. Boon, Patrick C. N. Rensen, Jose M. Llamas-Elvira, Jonatan R. Ruiz

**Affiliations:** ^1^PROFITH (PROmoting FITness and Health through Physical Activity) Research Group, Department of Physical Education and Sport, Faculty of Sport Sciences, University of Granada, Granada, Spain; ^2^Division of Endocrinology, and Einthoven Laboratory for Experimental Vascular Medicine, Department of Medicine, Leiden University Medical Center, Leiden, Netherlands; ^3^Servicio de Medicina Nuclear, Hospital Universitario Virgen de las Nieves, Granada, Spain; ^4^Servicio de Medicina Nuclear, Instituto de Investigación Biosanitaria (ibs. GRANADA), Granada, Spain; ^5^Department of Biochemistry and Molecular Biology II, Institute of Nutrition and Food Sciences, University of Granada, Granada, Spain; ^6^CIBEROBN, Biomedical Research Networking Center for Physiopathology of Obesity and Nutrition, Carlos III Health Institute, Madrid, Spain

**Keywords:** cooling vest, PET/CT scan, glucose uptake, thermal perception, body temperature

## Abstract

Brown adipose tissue (BAT) activity is induced when humans are exposed to cold. Therefore, cold exposure prior to the ^18^F-FDG-PET/CT scan is used as a tool to quantify BAT. Several cooling protocols, including fixed and personalized ones are currently in use. The aim of the present study was to determine the effect of a new personalized cooling protocol where the shivering threshold was measured on a separate day, on BAT volume and activity in young adults. A total of 47 adults (*n* = 28 women) aged 22 ± 2 years participated in the study. We determined participants' shivering threshold (visually and self-reported) using a water perfused cooling vest in an air-conditioned cold room. 48–72 h later, participants wore the cooling vest set at ~4°C above the shivering threshold for 60 min prior to injection of ^18^F-FDG and ~5°C above the shivering threshold for ~60 min after injection, until PET/CT scan. We quantified BAT following BARCIST 1.0 recommendations. We identified 40 participants (85%, *n* = 25 women) as PET+ and 7 (*n* = 3 women) as PET–. The PET+ group presented significantly higher BAT volume and activity than PET– group (all *P* < 0.05). PET+ women had higher BAT mean activity than PET+ men (SUV_mean_: 5.0 ± 1.6 vs. 3.6 ± 0.9 g/ml respectively, *P* = 0.003), and there were no significant sex differences in BAT volume (*P* = 0.161). A total of 9 out of 47 participants did not shiver during the shivering threshold test. Our findings are similar to previous cold-stimulated human BAT studies; therefore, we conclude that our personalized cooling protocol is able to activate BAT in young adults.

## Introduction

Obesity prevalence has increased exponentially during the last decades, and estimations indicate that global obesity prevalence will reach 18% in men and surpass 21% in women by 2025 (NCD Risk Factor Collaboration (NCD-RisC), 2016). Obesity is associated with a number of conditions and pathologies including insulin resistance, dyslipidaemia, type 2 diabetes, and cardiovascular diseases (Ng et al., 2014).

Brown adipose tissue (BAT) has the ability to oxidise glucose and lipids and to dissipate energy as heat which makes it an attractive target for anti-obesity and related comorbidities (Cannon and Nedergaard, 2004). BAT is mainly regulated by the sympathetic nervous system to defend core body temperature when mammals are exposed to temperatures below thermoneutrality (Cannon and Nedergaard, 2004; Boon and van Marken Lichtenbelt, 2015; Chechi et al., 2017). In 2009, a number of human studies showed that BAT is both present and thermogenically active in adults (Cypess et al., 2009; Saito et al., 2009; van Marken Lichtenbelt et al., 2009; Virtanen et al., 2009).

To date, the gold standard to quantify human BAT volume and activity is ^18^F-fluorodeoxyglucose (^18^F-FDG) positron emission tomography-computed tomography (PET/CT) (Nedergaard et al., 2007). Participants should be exposed to cold for a minimum of ~60 min prior to the injection of ^18^F-FDG and for ~60 min after the injection to maximize BAT activity and thus ^18^F-FDG uptake by the tissue (Chen et al., 2016). However, studies have used different cooling protocols to activate human BAT (Brychta and Chen, 2016; Chen et al., 2016). Whereas, some used cold exposure to a fixed and predefined temperature for all participants (Cypess et al., 2009), others used personalized cooling protocols, where the temperature is adjusted to the individual's shivering threshold (Bakker et al., 2014; van der Lans et al., 2014; Hanssen et al., 2016). A fixed protocol could induce sub-maximal non-shivering thermogenesis, whereas the personalized cooling protocol is likely to induce maximal non-shivering thermogenesis (van der Lans et al., 2014; Martinez-Tellez et al., 2017). Moreover, several authors applied different methodologies to induce cold stimuli including ice-blocks (Yoneshiro et al., 2011), cooling vests (Sanchez-Delgado et al., 2015), cooling blankets (Bakker et al., 2014), and air conditioning (Stahl et al., 2017), among others (Brychta and Chen, 2016), in combination with fixed or personalized cooling protocols. Therefore, the effect of these multiple combinations on the activation of human BAT is unknown.

Recently, an expert panel recommended the use of personalized cooling protocols to quantify BAT in humans, especially after an intervention that is expected to change BAT volume or activity (Chen et al., 2016). Brown Adipose Reporting Criteria in Imaging STudies (BARCIST 1.0) (Chen et al., 2016) recommendations include (i) determination of the shivering threshold and (ii) cold exposure at a relative shivering threshold temperature of the individual for 2 h prior to the PET/CT scan (Chen et al., 2016) on the same day, as other authors have done previously (Bakker et al., 2014; van der Lans et al., 2014; Hanssen et al., 2016). Therefore, the main difference between these studies and the present study, is that we measured the shivering threshold 48–72 h before to perform the PET/CT scan, in order to avoid excessive cold stress during BAT measurements.

The aim of the present study was to determine the effect of a novel personalized cooling protocol where the shivering threshold was measured on a separate day, on BAT volume and activity in young adults.

## Materials and methods

A total of 47 white Caucasian young adults (*n* = 28 women) aged 22 ± 2 years participated in the study (Table 1). Participants were enrolled in the ACTIBATE study (Sanchez-Delgado et al., 2015), an exercise-based randomized controlled trial (ClinicalTrials.gov ID:NCT02365129). All participants were healthy, sedentary (<20 min physical activity on <3 days/week), non-smokers, had no family history of type 2 diabetes, and did not take any medication that could influence the cardiovascular or thermoregulatory responses to cold exposure. The study protocol and informed consent were performed in accordance with the Declaration of Helsinki (revision of 2013). The study was approved by the Human Research Ethics Committee of the University of Granada (n°924) and of the Servicio Andaluz de Salud (Centro de Granada, CEI-Granada). The evaluations were performed in four waves of ~12 participants each, from 15th October to 28th November, 2015 in Granada (Spain).

**Table 1:** Characteristics of participants by sex.

	**Men (n = 19)**	**Women (n = 28)**	**P**
Age (years)	22.1 ± 2.1	21.9 ± 1.8	0.760
BMI (kg/m^2^)	27.6 ± 5.2	23.2 ± 3.6	**0.001**
Fat mass (kg)	25.9 ± 9.9	23.2 ± 7.9	0.294
Lean mass (kg)	53.4 ± 7.7	35.0 ± 5.1	**<0.001**
Fasting glucose (mmol/l)	4.9 ± 0.4	9.3 ± 6.3	0.243
Fasting insulin (μIU/ml)	4.8 ± 0.4	8.3 ± 4.8	0.569

*Data are presented as mean and standard deviation. BMI, Body mass index. P for sex comparisons. The bold values indicate statistically significant differences*.

### Previous conditions to the study days

Participants arrived to the research center by bus or by car, and in fasting conditions (at least 6 h). They were advised to (i) sleep as usual, (ii) refrain from any moderate (for 24 h) or vigorous (for 48 h) physical activity, and (iii) to not consume alcoholic or stimulant beverages (for 6 h), or drugs affecting peripheral circulation (24 previous hours). We encouraged them to drink room-temperature water before the tests, ~1 l before the shivering threshold test (STT) and ~2 l before the PET/CT scan.

### Shivering threshold test

**Figure 1:**
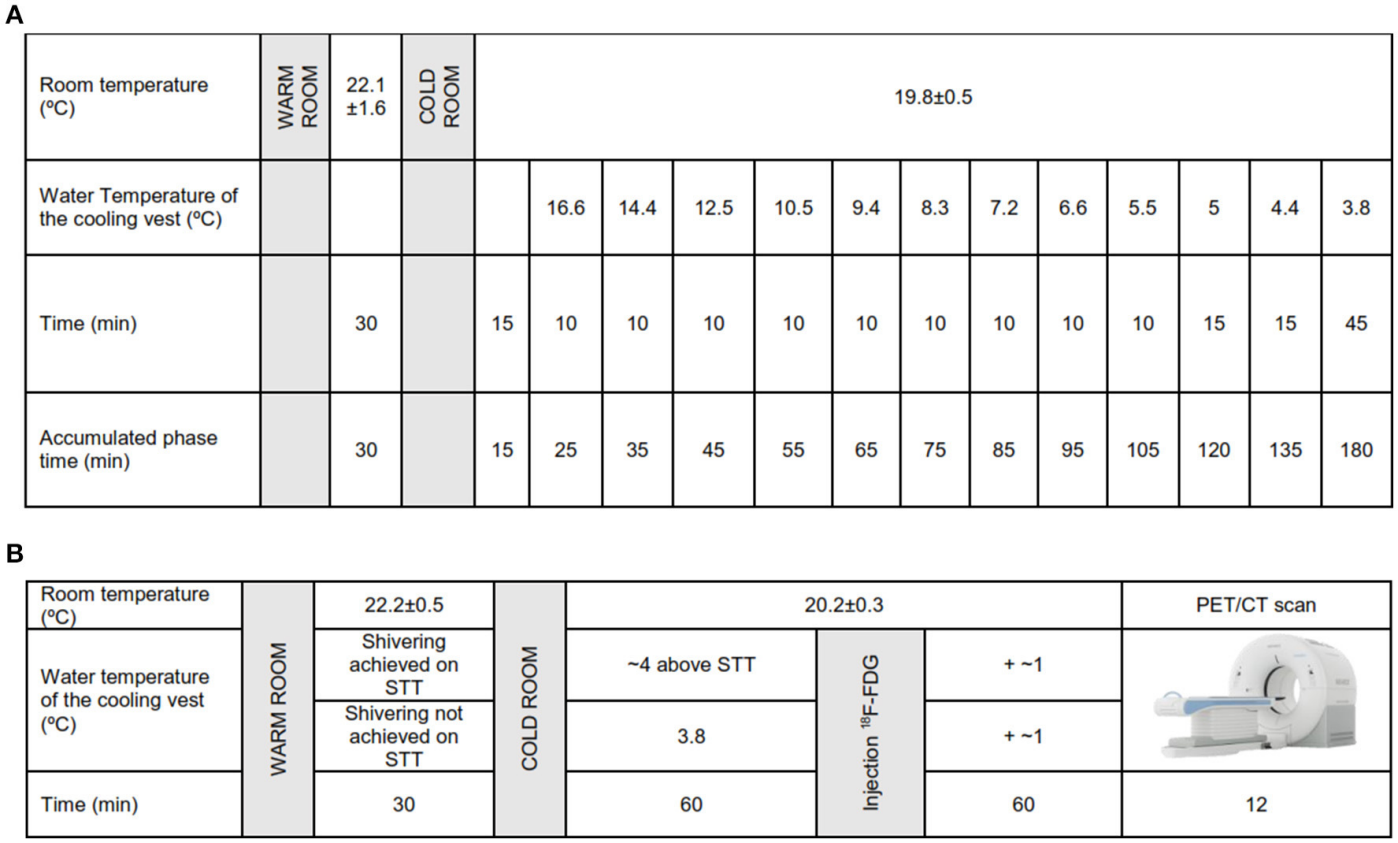
**(A)** Shivering threshold test (STT) protocol. **(B)** Personalized cooling protocol prior to ^18^F-fluorodeoxyglucose (^18^F-FDG)-Positron emission tomography/computed tomography (PET/CT) scan.

We conducted the STT 48–72 h before applying 2 h of personalized cold exposure prior to the PET/CT scan. Upon arrival to the research center, all participants confirmed that they had followed the pre-study instructions. They emptied their bladders, dressed up with standardized clothes [sandals, T-shirt and shorts, clo-value: 0.20 (ISO-standard 9920 Ergonomics of the thermal environment estimation of thermal insulation and water vapour resistance of a clothing ensemble, 2009)], and entered into a warm room (22.1 ± 1.6°C) where they remained seated for 30 min (Figure 1A). Participants received detailed information and instructions about the STT protocol. Afterwards, they entered into a cold room (19.8 ± 0.5°C) where they remained seated in a chair for 15 min, and were not allowed to stand up, move, rub, or cover their bodies. Then, participants dressed up with a temperature-controlled water perfused cooling vest (Polar Products Inc., Ohio, USA), which covers the clavicular region, as well as the chest, abdominals, and the back. Water temperature was set at 16.6°C and decreased progressively every 10 min until 5.5°C (Figure 1A). If participants did not report shivering and researchers did not observe it either, we decreased water temperature by 0.6°C every 15 min until 3.8°C. At this stage, if shivering had not occurred, participants remained in the cold room for another 45 min, after which the test was finished (Figure 1A). Women were kindly asked to tie up their hair to reduce hair insulation over the neck and shoulders. We determined shivering visually and by asking the participants if they were experiencing shivering. We had previously observed that both self-reported and visual inspection of shivering concurred with muscle activity measured by EMG in an independent group of six young adults (unpublished observations).

We recorded whole-body, clavicular, and hands thermal sensation at the end of the warm period and at the end of the STT using a continuous 7-points thermal sensation interval scale (American Society of heating, refrigerating, and air conditioning engineers, ASHRAE) (American Society of Heating Refrigerating and Air-Conditioning Engineers, 2005; Paliaga et al., 2013). Participants also reported the subjective perception of shivering in a numeric rate scale (NRS) where 0 refers to “I am not shivering” and 10 refers to “I am shivering a lot.”

### Personalized cooling protocol prior to PET/CT scan

Participants confirmed that they had followed all pre-study conditions and were invited to empty their bladders and dressed up with the standardized clothes (the same as the STT day). They stayed in a warm room for 30 min (22.2 ± 0.5°C), after which they entered into a cold room (20.2 ± 0.3°C). As in the STT day, participants wore the same temperature controlled water perfused cooling vest (Polar Products Inc., Ohio, USA) for 60 min set at ~4°C above the temperature that caused the onset of shivering. If the participant did not report shivering in the STT, water temperature was settled at 3.8°C, similar to other personalized cooling protocols (Bakker et al., 2014; van der Lans et al., 2014). Participants were instructed to immediately inform the researchers if they experienced shivering at any time. When shivering was reported, we increased the water temperature by 1°C and provided a bathrobe for 2 min until shivering disappeared. After 60 min of personalized cold exposure, we administrated an intravenous ^18^F-FDG injection (185 MBq; ~2.78 MBq/kg), and we increased water temperature by ~1°C. This temperature was kept constant for another 60 min (Figure 1B). After 2 h of personalized cold exposure, participants went into the PET/CT scan (Siemens Biograph 16 PET/CT, Siemens, Germany). For the CT acquisition a peak kilovoltage of 120 was applied, while for the PET acquisition a scan time of 6 min per bed position was set. In total, 2 bed positions were scanned from *atlas vertebrae* to *thoracic vertebrae 4*. Participants reported their thermal sensation (by ASHRAE-scales) and the subjective level of shivering (NRS) in the warm room and at the end of the cooling exposure period in the same way as in the STT day.

### PET/CT analysis

The PET/CT scans were analyzed using Beth Israel plugin for FIJI (Cypess et al., 2009) software (http://sourceforge.net/projects/bifijiplugins/ Schindelin et al., 2012). We calculated the standardized uptake value (SUV) as [^18^F-FDG uptake (kBq/mL)/(injected dose [kBq]/patient weight [g])]. SUV threshold was calculated as SUV≥ 1.2/(lean body mass/body mass) (Chen et al., 2016). We applied a fixed range of Hounsfield units (HU, −190 to −10) (Chen et al., 2016). PET/CT images were carefully analyzed by BMT with the supervision of a nuclear doctor (JML) and with an expert on the field of human BAT (MRB).

The region of interest (ROI) was semi-automatically outlined from *atlas vertebrae* (Cervical 1) to *thoracic vertebrae* 4. We determined BAT volume, SUV_mean_, BAT metabolic activity, SUV_peak_, and SUV_max_ according to BARCIST 1.0 recommendations (Hasenclever et al., 2014; Chen et al., 2016). Furthermore, we also considered as BAT-depots all pixels that achieved the predefined thresholds of SUV and HU (Chen et al., 2016). The PET/CT scans were visually and carefully examined to detect ^18^F-FDG uptake in BAT-specific depots. Participants were categorized as PET+ when BAT volume was ≥5 ml and ^18^F-FDG uptake was clearly apparent, and as PET- when BAT volume was <5 ml and there were no signs of cold-stimulated ^18^F-FDG uptake in the BAT region (Gifford et al., 2016). Body composition was measured on a separate day by Dual Energy X-ray Absorptiometry (HOLOGIC, QDR 4500W) (Sanchez-Delgado et al., 2015).

### Statistical analysis

Data are presented as mean ± standard deviation, unless otherwise stated. We used a one-way analysis of variance (ANOVA) to test differences in body composition and in BAT outcomes by cold-stimulated ^18^F-FDG uptake by BAT (PET+ vs. PET–) and by sex (men vs. women). Categorical variables (sex and weight status) were compared using the *X*^2^ test. We used a paired *t*-test to study differences on self-reported thermal sensations and shivering between the STT and the personalized cooling protocol prior to the PET/CT scan in both warm period and at the end of the cooling exposure. We estimated the effect size as previously reported (Cohen, 1992). We established as dependent variable BAT-related outcomes and as independent variable sex. We found a medium effect for BAT volume (d = 0.45), a moderate effect for SUV_peak_ (d = 0.63), and SUV_max_ (d = 0.69) and a large effect for SUV_mean_ (d = 1.08). Moreover, when we established as independent variable PET+ or PET– groups the effect size of BAT-related outcomes increased even more (all d ≥ 2.07). Analyses were conducted using the Statistical Package for Social Sciences (SPSS, v. 22.0, IBM SPSS Statistics, IBM Corporation) and the level of significance was set to <0.05.

## Results

### Characteristics of the participants

There were no differences of age or fat mass between sexes (*P* > 0.2), yet men had a higher BMI and lean mass than women (all *P* ≤ 0.001) (Table 1). However, we did not find significant differences in fasting glucose and insulin levels between sexes (*P* = 0.243 and *P* = 0.569, respectively).

### Cooling protocol

A total of 38 participants (*n* = 25 women) reached shivering during the STT and 9 participants (*n* = 3 women) did not. There were no significant differences in BAT-related outcomes between both groups (all *P* > 0.3, data not shown). There were no significant sex differences in mean water vest temperature at the end of STT (5.8 ± 2.2 vs. 6.4 ± 1.8°C, men and women, respectively, *P* = 0.875) or time until the end of STT (119.7 ± 32.1 vs. 108.4 ± 31.9 min, men and women, respectively, *P* = 0.716).

**Figure 2:**
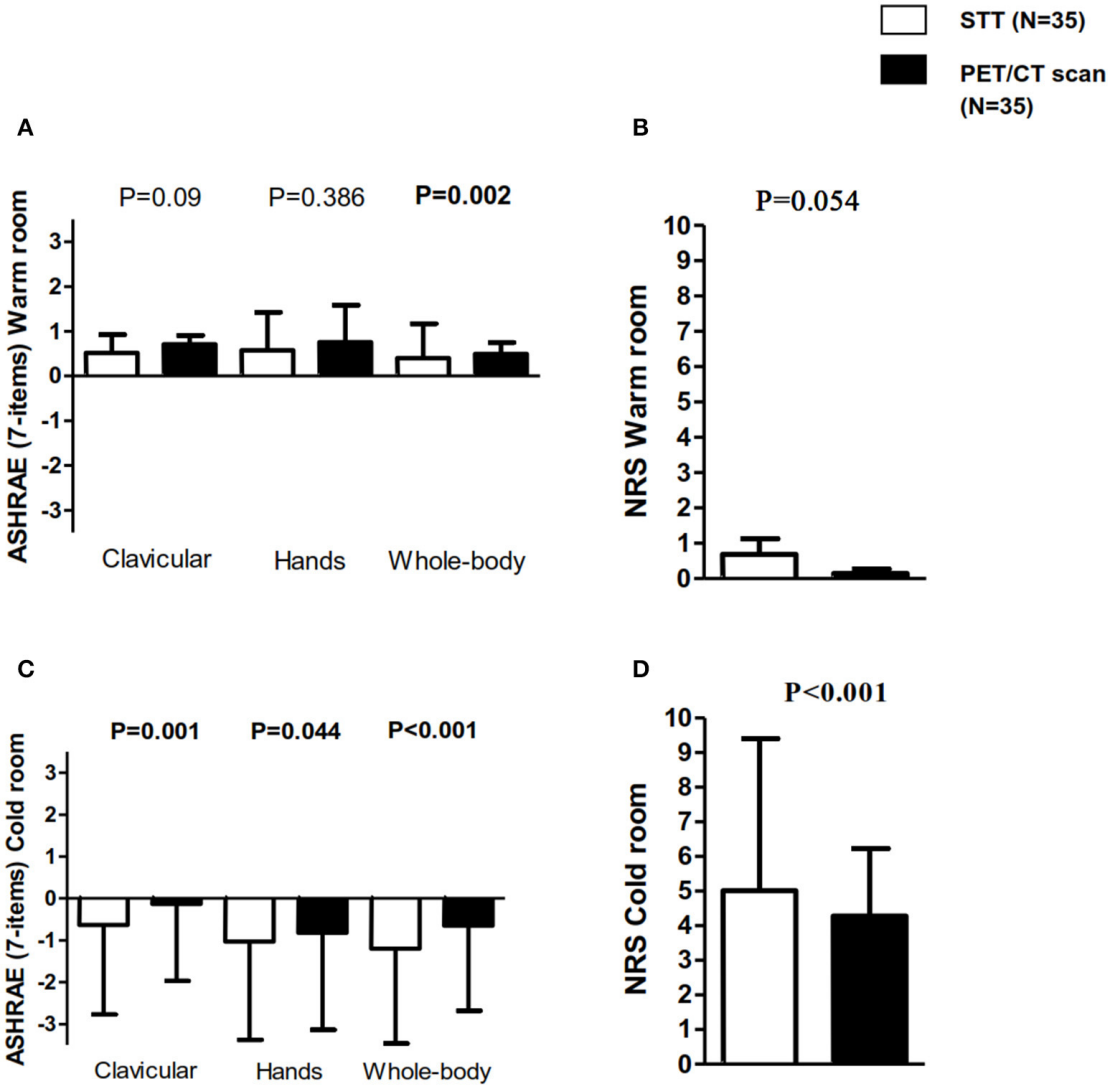
Self-report thermal sensation (in the clavicular, hands, and whole-body zones) and subjective perception of shivering in the shivering threshold test (STT) and in the personalized cooling protocol before ^18^F-fluorodeoxyglucose (^18^F-FDG)-Positron emission tomography/computed tomography (PET/CT) scan. **(A)** American Society of heating, refrigerating and air conditioning engineers (ASHRAE) scales of 7 points: −3 = cold, 0 = neutral, and 3 = hot in the warm room. **(B)** Numeric rate scale (NRS): 0 = “I not shivering” and 10 = “I am shivering a lot” in the warm room. **(C)** ASHRAE scales of 7 points in the cold room. **(D)** NRS in the cold room. All data are represented as mean and standard deviation. P for STT vs. PET/CT comparisons.

Data on self-reported thermal sensation was available in 35 participants. The self-reported thermal sensation (assessed by ASHRAE scales) in the clavicular and hands zones as well as the subjective perception of shivering (assessed by NRS) were similar at the end of the warm period in both the STT and in the personalized cooling protocol before the ^18^F-FDG-PET/CT scan, see Figure 2B. However, whole-body thermal sensation was slightly higher (i.e., warmer, *P* = 0.002) at the end of the warm period on the STT day (Figure 2A). The self-reported thermal sensation and the subjective perception of shivering were significantly higher at the end of the STT than in the personalized cooling protocol before the ^18^F-FDG-PET/CT scan (i.e., cooler, all *P* ≤ 0.05, see Figures 2C,D).

### ^18^F-FDG-PET/CT measurements

**Figure 3:**
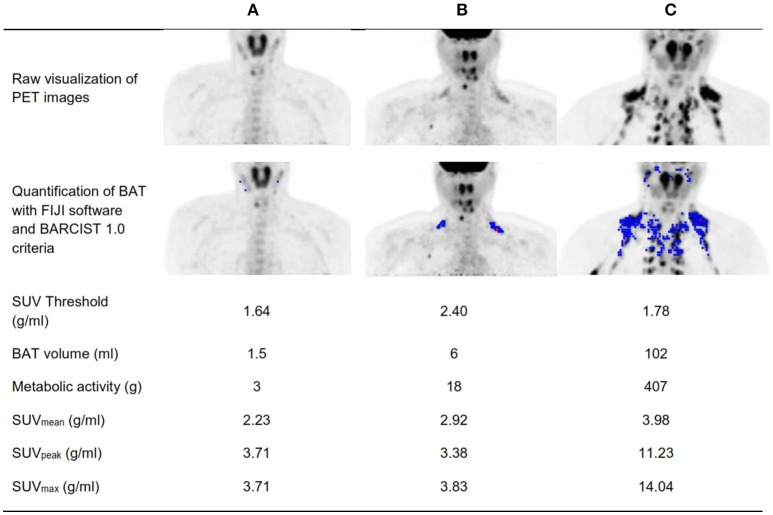
Visual determination of a positron emission tomography with a participant positive for ^18^F-FDG uptake (PET+) and a participant negative for ^18^F-FDG uptake (PET–). **(A)** A PET- participant with negligible cold-stimulated brown adipose tissue (BAT) ^18^F-FDG uptake. **(B)** A PET+ participant with the lowest cold-stimulated BAT ^18^F-FDG uptake. **(C)** A PET+ participant with high cold-stimulated BAT ^18^F-FDG uptake. BMI, Body mass index; SUV, Standardized uptake value.

We identified 40 participants (25 women) as PET+ and 7 (3 women) as PET–. Figure 3A shows an example of a PET– participant with negligible cold-stimulated BAT ^18^F-FDG uptake a PET+ participant with the low cold-stimulated BAT ^18^F-FDG uptake (Figure 3B), and a PET+ participant with high cold-stimulated BAT ^18^F-FDG uptake (Figure 3C). Mean water vest temperature at the end of STT and time up to the end of STT was similar in the PET+ and PET– groups (6.1 ± 1.9 vs. 6.7 ± 2.1°C, respectively, *P* = 0.994; and 115.2 ± 30.1 vs. 100.3 ± 42.5 min, respectively, *P* = 0.291).

There were no significant differences on self-reported thermal sensation between PET+ and PET– group (all *P* > 0.1, data not shown).

Table 2 shows the participants' characteristics categorized as PET+ and PET–. There were no statistical significant differences with respect to age, sex, or body composition between groups (all *P* > 0.05) (Table 2). The PET+ group had significantly higher SUV threshold (*P* = 0.035), BAT volume (*P* < 0.001), BAT metabolic activity (*P* < 0.001), SUV_mean_ (*P* < 0.001), SUV_peak_ (*P* < 0.001), and SUV_max_ (*P* < 0.001) than their PET– counterparts (Table 2).

**Table 2:** Characteristics of participants by positron emission tomography with positive glucose uptake (PET+) vs. negative glucose uptake (PET–).

	**PET+ (n = 40)**	**PET– (n = 7)**	**P**
Age (years)	21.8 ± 1.9	23.1 ± 1.3	0.086
Sex (n,%)			0.329
Men	15 (38)	4 (57)	
Women	25 (62)	3 (43)	
BMI (kg/cm^2^)	25.3 ± 5.0	23.2 ± 2.6	0.297
Weight status (n,%)			0.276
Normal-weight	22 (55)	6 (85.7)	
Overweight	11 (27.5)	1 (14.2)	
Obese	7 (17.5)	0	
Fat mass (kg)	25.2 ± 8.8	19.3 ± 6.6	0.101
Fat mass index (kg/cm^2^)	9.0 ± 3.0	6.7 ± 2.9	0.072
Lean mass (kg)	41.9 ± 11.3	45.3 ± 9.0	0.457
Lean mass index (kg/cm^2^)	14.9 ± 3.3	15.4 ± 2.2	0.727
SUV threshold (g/ml)	2.1 ± 0.2	1.9 ± 0.3	**0.035**
BAT volume (ml)	96 ± 58	1.5 ± 1.7	**<0.001**
Metabolic activity (g)	467 ± 344	3.3 ± 3.6	**0.001**
SUV_mean_ (g/ml)	4.5 ± 1.5	1.9 ± 0.9	**<0.001**
SUV_peak_ (g/ml)	11.8 ± 6.1	2.7 ± 1.4	**<0.001**
SUV_max_ (g/ml)	14.9 ± 7.6	2.9 ± 1.5	**<0.001**

*Data are presented as mean and standard deviation, unless otherwise stated. P for PET+ vs. PET– comparisons. BAT, Brown adipose tissue; BMI, Body mass index; SUV, Standardized uptake value. The bold values indicate statistically significant differences*.

**Figure 4:**
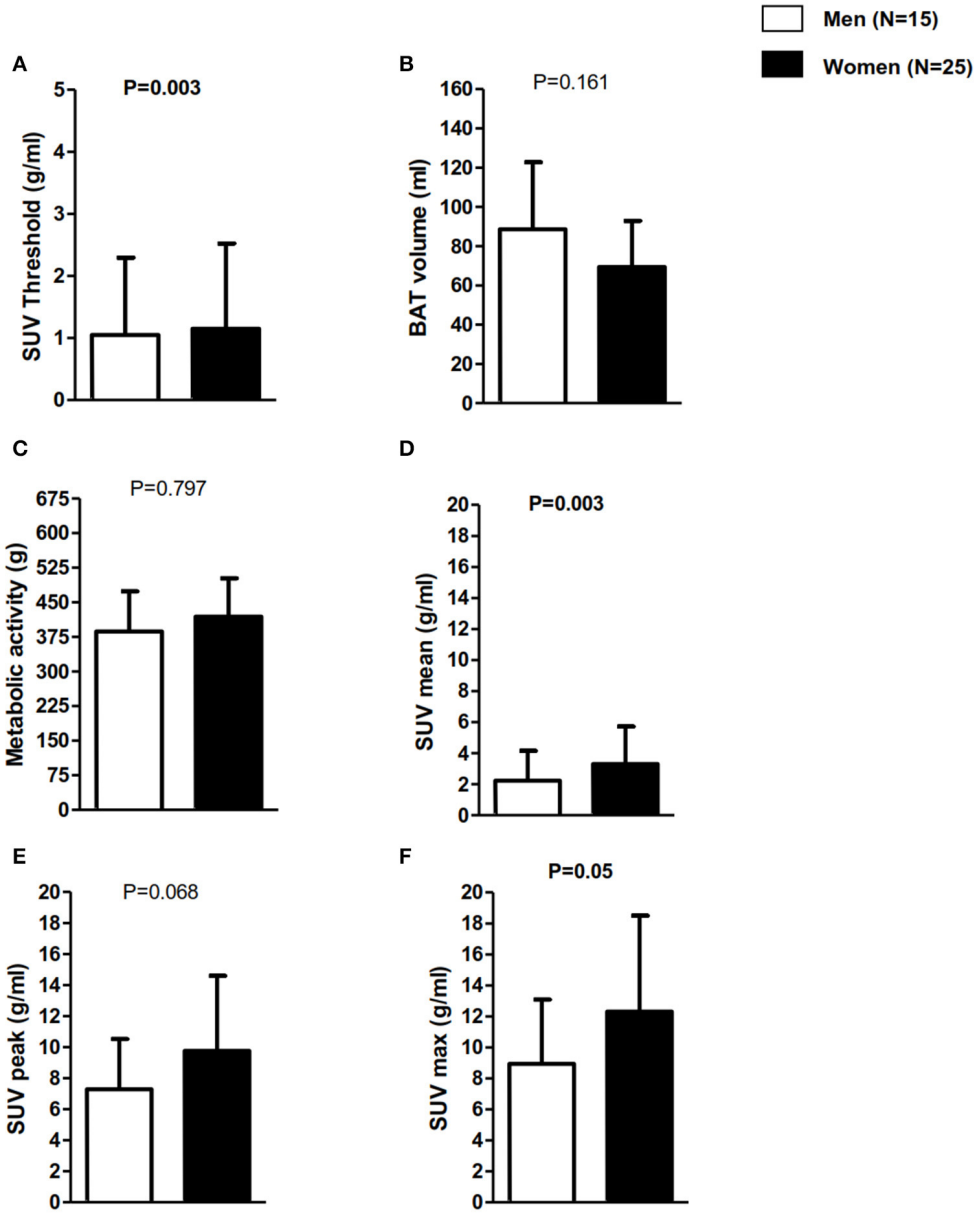
Brown adipose tissue-related outcomes in positron emission tomography in participants positive for ^18^F-FDG uptake (PET+) classified in men vs. women. Data are mean and standard deviation. **(A)** SUV individualized threshold to lean body mass percentage. **(B)** BAT volume in mililiters. **(C)** BAT metabolic activity (BAT volume x SUV mean. **(D)** BAT activity represented as SUVmean (g/ml). **(E)** BAT activity represented as SUVpeak (g/ml). **(F)** BAT activity represented as SUVmax (g/ml). P for sex comparisons. BAT, Brown adipose tissue; BMI, Body mass index; SUV, Standardized uptake value.

Within the PET+ group, SUV threshold was higher in women than in men (2.1 ± 0.2 vs. 1.9 ± 0.2 g/ml, respectively, *P* = 0.003, Figure 4A). Women also presented higher SUV_mean_ (5.0 ± 1.6 vs. 3.6 ± 0.9 g/ml; *P* = 0.003, Figure 4D), slightly higher SUV_peak_ (13.2 ± 6.4 vs. 9.6 ± 5.0 g/ml; *P* = 0.068, Figure 4E), and higher SUV_max_ (16.7 ± 7.9 vs. 11.9 ± 6.0 g/ml; *P* = 0.05, Figure 4F) than men. There were no sex differences in BAT volume (86 ± 53 vs. 113 ± 65 ml, women and men, respectively, *P* = 0.161, Figure 4B) or BAT metabolic activity (478 ± 361 vs. 448 ± 325 g, respectively, *P* = 0.797, Figure 4C).

## Discussion

In the present study we describe a new personalized cooling protocol in young adults that follows the BARCIST 1.0 recommendations to determine BAT volume and activity (Chen et al., 2016) where the shivering threshold was determined on a separate day. A total of 40 out of 47 (87%) participants were identified as PET+, and seven participants were identified as PET– even after 2 h of personalized cold-exposure prior to the PET/CT scan. As expected, PET+ participants had higher BAT volume, BAT metabolic activity, SUV_mean_, SUV_peak_, and SUV_max_ than PET–. Of note, SUV threshold was higher in the PET+ group due to the fact that this group presented slightly higher levels of fat mass. PET+ women had higher BAT activity than PET+ men, whereas there were no sex differences in BAT volume.

To our knowledge, this is the first study that applied a 2-h personalized cooling protocol prior to the PET/CT scan while determining the shivering threshold on a separate day (48–72 h before). Of note is that all the personalized cooling protocols conducted the STT and the 2-h cold exposure prior to the PET/CT scan (Vijgen et al., 2011, 2013; Orava et al., 2013; Vosselman et al., 2013; Boon et al., 2014; Hanssen et al., 2015a; Lee et al., 2016; McCallister et al., 2017) on the same day. This design could be a burden as well as an extra cold-stress for participants, such as older (Kindred et al., 2016) or unhealthy (Cao et al., 2014) participants. Moreover, personalized cooling protocols with shorter cold exposures could be useful to study the effect of certain drugs or in other population such as children, older people or unhealthy, to activate BAT. Retrospective studies showed that without prior cooling, BAT depots could be detected by PET/CT scan in ~6% of adults (Lee et al., 2011). Nevertheless, when cooling exposure is applied prior to the PET/CT scan, the prevalence of PET+ is 20–31% in obese (Orava et al., 2013) and 40–100% in lean healthy adults (van der Lans et al., 2014, 2016). In the present study, the prevalence of PET+ in young adults was 85%, which concurs with previous observations. More studies are needed to elucidate which cooling protocol is more efficient in terms of BAT activation and that at the same time causes participants less discomfort.

Recently, an expert panel launched a set of recommendations for conducting ^18^F-FDG-PET/CT experiments of human BAT, data analysis, and publication of results (Chen et al., 2016). They suggested the use of a fixed criteria of HU (−190,−10) and an individualized SUV threshold based on body composition. They recommended the use of a SUV threshold of ≥1.2 divided by the participant's lean body mass/body mass (Chen et al., 2016) because ^18^F-FDG uptake is higher for lean tissue than for white fat. Therefore, women are expected to have a higher SUV threshold than men because they have lower levels of lean body mass and higher levels of white fat, which was confirmed in our study (Figure 4A). However, studies that used personalized cooling protocols did not find differences in BAT volume and activity between sexes (Pfannenberg et al., 2010), whereas in studies in which a fixed cooling protocol was used, women had higher activity than men (Cypess et al., 2009). These discrepancies could be based on different methodological protocols for BAT quantification (Martinez-Tellez et al., 2017). Nevertheless, we applied a personalized cooling protocol and we found that women had higher activity than men. This finding could be based on the fact that women were leaner than men (see Table 1). Nevertheless, more studies are warranted to elucidate actual sex differences.

The levels of SUV_max_ observed in our study are in agreement with those reported by Bakker et al. (2014) in Caucasians and south Asians (SUV_max_: 15 g/ml), and by Hanssen et al. (2015b) in lean participants (SUV_max_: 15.9 ± 5.8 g/ml). Of note is that both studies applied a personalized cooling protocol. Taken together, these findings suggest that applying just 2 h of personalized cold exposure prior to the PET/CT scan induces similar BAT activation in young adults than when both shivering threshold and 2 h of cold exposure prior to the PET/CT scan are conducted on the same day. Levels of SUV_max_ might be less influenced by the methods used to quantify BAT, and it therefore allows between study comparisons. However, other BAT-related outcomes cannot be directly compared across studies without assuming that differences might be largely explained by the methodology used to quantify or activate BAT (Martinez-Tellez et al., 2017). Therefore, the present study showed that a short cold exposure can induce BAT activation to a similar extent as longer cold exposures.

The observed subjective thermal sensations observed in our study are similar to other studies that used the same (Schellen et al., 2013; Vosselman et al., 2014) or different instruments (e.g., visual analog scales) (van der Lans et al., 2013; Vosselman et al., 2014; Hanssen et al., 2015b; Yoneshiro et al., 2016). As expected, participants had a higher thermal sensation and higher levels of subjective shivering during the STT, because the aim of this test was to produce shivering, while the aim of the personalized cooling protocol prior to the ^18^F-FDG-PET/CT scan was to produce maximal non-shivering thermogenesis.

### Limitations

The results of this study should be considered with caution. We performed the PET/CT scan from *cerebellum* to *thoracic vertebrae 4*, and we may have therefore missed BAT depots localized in other areas. Nevertheless, most of the BAT depots detected in humans are localized in the areas covered by our scan (Nedergaard et al., 2007; Leitner et al., 2017). The study was conducted in young men and women, and we do not know whether these results apply to older adults. It would be interesting to compare the results on BAT parameters as well as skin and core temperature in humans applying different cooling protocols, to validate if different cold exposures are able to activate human BAT. However, this is not possible due to the high radiation associated to the ^18^F-FDG-PET/CT scans. Furthermore, future studies should analyse the relationship between the BAT volume and activity and time to shivering. Finally, given the limited sample size of this study, which is therefore prone to type 2 errors, additional studies are needed to better understand the differences between PET+ and PET– groups.

## Conclusion

Our cooling protocol is able to activate BAT in young adults. The novelty of our protocol resides in the fact that we applied just 2 h of personalized cold exposure prior to PET/CT scan while the shivering threshold was determined on a separate day (48–72 h before).

## Author contributions

Conception and design of research: BM, GS, JA, JL, and JR. BM, GS, YG, JA, WM, MM, JO, MB, PR, JL, and JR performed the experiments; BM, GS, YG, WM, MM, JO, MB, PR, JL, and JR analyzed the data; BM, GS, YG, JA, WM, MM, JO, MB, PR, JL, and JR interpreted the results; BM and JR prepared the figures and drafted manuscript; BM, GS, YG, JA, WM, MM, JO, MB, PR, JL, and JR critically revised the manuscript and approved the final version.

## Prior presentation

Parts of this study were presented as a poster presentation at the EMBO Workshop: Brown adipose tissue. Sitges, Spain, 24–27 May 2017.

### Conflict of interest statement

The authors declare that the research was conducted in the absence of any commercial or financial relationships that could be construed as a potential conflict of interest.
